# The genome sequence of the Festoon, *Apoda limacodes* (Hufnagel, 1766)

**DOI:** 10.12688/wellcomeopenres.18747.1

**Published:** 2023-01-12

**Authors:** Gavin R. Broad, Natural History Museum Genome Acquisition Lab, Darwin Tree of Life Barcoding collective, Wellcome Sanger Institute Tree of Life programme, Wellcome Sanger Institute Scientific Operations: DNA Pipelines collective, Tree of Life Core Informatics collective, Darwin Tree of Life Consortium

**Affiliations:** 1 Department of Life Sciences, Natural History Museum, London, UK

**Keywords:** Apoda limacodes, the Festoon, genome sequence, chromosomal, Lepidoptera

## Abstract

We present a genome assembly from an individual male *Apoda limacodes* (the Festoon; Arthropoda; Insecta; Lepidoptera; Limacodidae). The genome sequence is 800 megabases in span. Most of the assembly is scaffolded into 25 chromosomal pseudomolecules, including the assembled Z sex chromosome. The mitochondrial genome has also been assembled and is 15.4 kilobases in length.

## Species taxonomy

Eukaryota; Metazoa; Ecdysozoa; Arthropoda; Hexapoda; Insecta; Pterygota; Neoptera; Endopterygota; Lepidoptera; Glossata; Ditrysia; Zygaenoidea; Limacodidae; *Apoda*; *Apoda limacodes* (Hufnagel, 1766) (NCBI:txid287200)

## Background

The larva of the Festoon, *Apoda limacodes*, is an odd caterpillar. It has no visible legs and no obvious body segmentation, hence its Latin name, ‘legless, slug-like’. The Festoon and the Triangle ( *Heterogenea asella*) are the only British species of Limacodidae, a cosmopolitan family of about 1,700, mainly tropical, described species. They are often called slug moths and many species have spectacular larvae, sometimes with stinging setae. Larvae of the Festoon are harmless, and the adults are relatively sombre in colour, although nothing else in Britain looks like them. Adults have a characteristic posture with the wings arranged in a tent-like fashion and the abdomen pointing upwards. Females lack the darker band across the middle of the wing.

Larvae of the Festoon feed mainly on oak and beech; caterpillars can be found most easily at night as they fluoresce under the beam of a UV torch. Adults come to light or can sometimes be seen flying in the canopy. As with other limacodids, instead of abdominal prolegs, there are sucker-like discs which enable the larvae of limacodids to seem to glide along the leaf surface. Adults are on the wing in June and July with larvae feeding from late summer then spending the winter as cocooned prepupae in the leaf litter, pupating the following spring, e.g. Henwood *et al.*, 2020.

Often associated with mature woodlands, the Festoon has increased significantly in range in recent years ( Fox *et al.*, 2014), although is still mainly a species of south-east England (NBN Atlas), but this probably follows a period of much reduced abundance. The larvae of *A. limacodes* support a suite of highly specialised parasitoid wasps (e.g., ( Shaw & Voogd, 2016) and one, *Sphinctus serotinus* Gravenhorst, was recently rediscovered in England after an apparent absence of over a century ( Thornhill, 2020).

This is the first genome for a species of the family Limacodidae, so will be invaluable in comparative genomics across Lepidoptera. As a family which includes several notable agricultural pests ( Cock *et al.*, 1987), a limacodid reference genome should be useful in studying the genetics of related pest species.

## Genome sequence report

**Figure 1.**
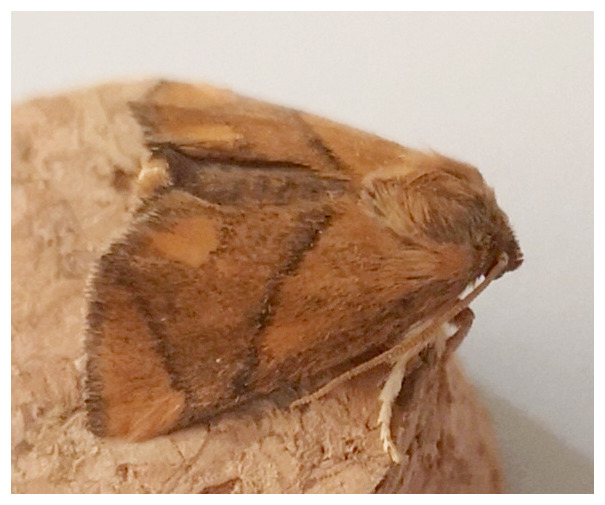
Image of the *Apoda limacodes* (ilApoLima1) specimen used for genome sequencing.

**Figure 2.**
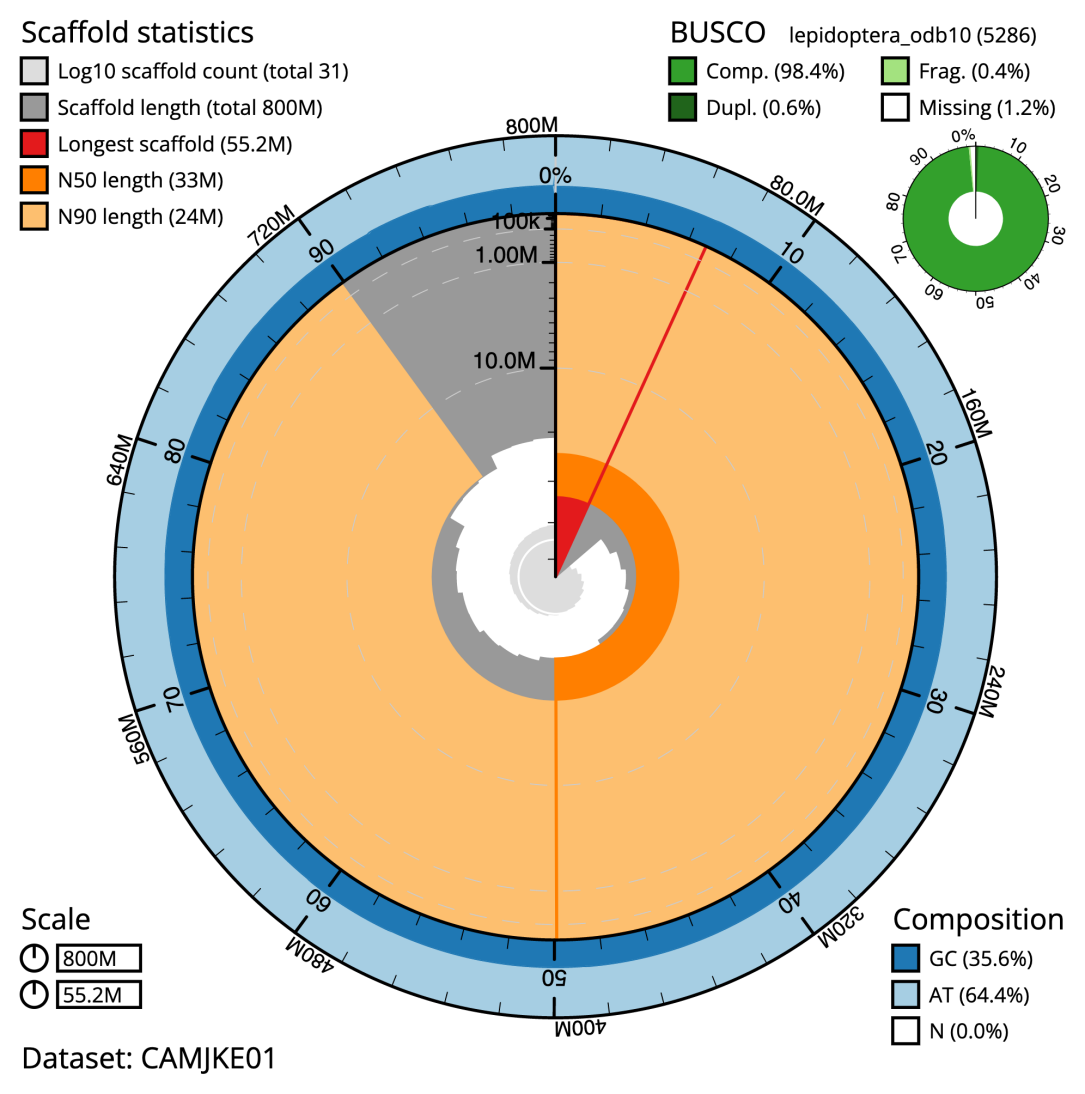
Genome assembly of *Apoda limacodes*, ilApoLima1.1: metrics. The BlobToolKit Snailplot shows N50 metrics and BUSCO gene completeness. The main plot is divided into 1,000 size-ordered bins around the circumference with each bin representing 0.1% of the 800,350,849 bp assembly. The distribution of sequence lengths is shown in dark grey with the plot radius scaled to the longest sequence present in the assembly (55,212,830 bp, shown in red). Orange and pale-orange arcs show the N50 and N90 sequence lengths (33,043,310 and 23,972,641 bp), respectively. The pale grey spiral shows the cumulative sequence count on a log scale with white scale lines showing successive orders of magnitude. The blue and pale-blue area around the outside of the plot shows the distribution of GC, AT and N percentages in the same bins as the inner plot. A summary of complete, fragmented, duplicated and missing BUSCO genes in the lepidoptera_odb10 set is shown in the top right. An interactive version of this figure is available at https://blobtoolkit.genomehubs.org/view/ilApoLima1.1/dataset/CAMJKE01/snail.

The genome was sequenced from a male *A. limacodes* ( Figure 1) collected from Tonbridge, UK. A total of 23-fold coverage in Pacific Biosciences single-molecule HiFi long reads was generated. Primary assembly contigs were scaffolded with chromosome conformation Hi-C data. Manual assembly curation corrected 6 missing or mis-joins, reducing the scaffold number by 3.13%. The final assembly has a total length of 800.4 Mb in 31 sequence scaffolds with a scaffold N50 of 33.0 Mb ( Table 1). Most (99.98%) of the assembly sequence was assigned to 25 chromosomal-level scaffolds, representing 24 autosomes, and the Z sex chromosome. Chromosome-scale scaffolds confirmed by the Hi-C data are named in order of size ( Figure 2– Figure 5; Table 2). The assembly has a BUSCO v5.3.2 ( Manni *et al.*, 2021) completeness of 98.4% (single 97.8%, duplicated 0.6%) using the OrthoDB v10 lepidoptera reference set. While not fully phased, the assembly deposited is of one haplotype. Contigs corresponding to the second haplotype have also been deposited.

**Table 1.** Genome data for *Apoda limacodes*, ilApoLima1.1.

Project accession data		
Assembly identifier	ilApoLima1.1	
Species	*Apoda limacodes*	
Specimen	ilApoLima1	
NCBI taxonomy ID	287200	
BioProject	PRJEB54603	
BioSample ID	SAMEA11025013	
Isolate information	Head/thorax tissue (HI-C and PacBio sequencing)	
Assembly metrics *		*Benchmark*
Consensus quality (QV)	61.4	*≥ 50*
*k*-mer completeness	100%	*≥ 95%*
BUSCO **	C:98.4%[S:97.8%,D:0.6%], F:0.4%,M:1.2%,n:5,286	*C ≥ 95%*
Percentage of assembly mapped to chromosomes	99.98%	*≥ 95%*
Sex chromosomes	Z chromosome	*localised homologous pairs*
Organelles	Mitochondrial genome assembled	*complete single alleles*
Raw data accessions		
PacificBiosciences SEQUEL II	ERR9959164	
Hi-C Illumina	ERR9930694	
Genome assembly		
Assembly accession	GCA_946406115.1	
*Accession of alternate haplotype*	GCA_946409105.1	
Span (Mb)	800.4	
Number of contigs	90	
Contig N50 length (Mb)	15.9	
Number of scaffolds	31	
Scaffold N50 length (Mb)	33.0	
Longest scaffold (Mb)	55.2	

* Assembly metric benchmarks are adapted from column VGP-2020 of “Table 1: Proposed standards and metrics for defining genome assembly quality” from ( Rhie *et al.*, 2021).

** BUSCO scores based on the lepidoptera_odb10 BUSCO set using v5.3.2. C = complete [S = single copy, D = duplicated], F = fragmented, M = missing, n = number of orthologues in comparison. A full set of BUSCO scores is available at https://blobtoolkit.genomehubs.org/view/ilApoLima1.1/dataset/CAMJKE01/busco.

**Figure 3.**
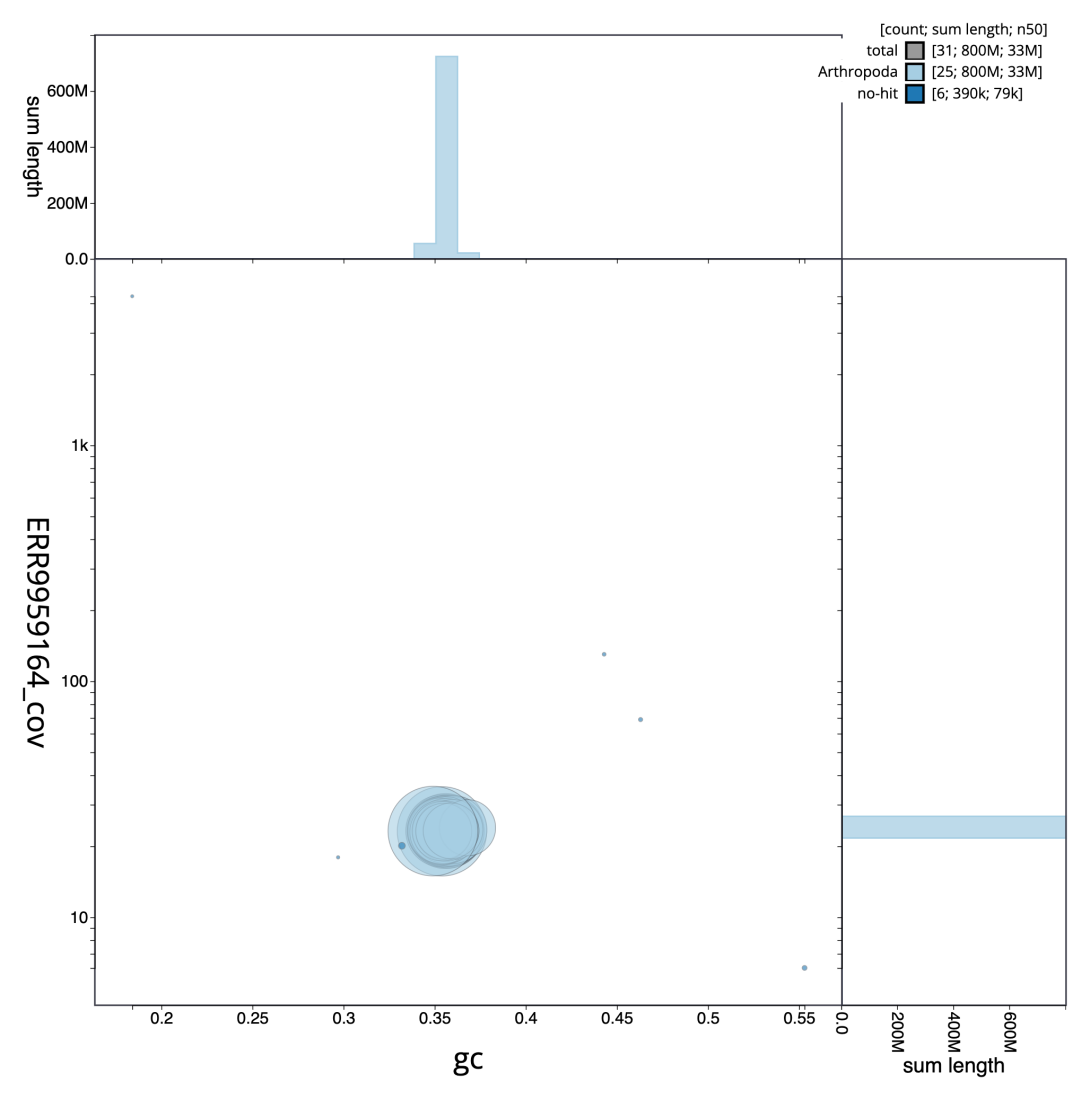
Genome assembly of *Apoda limacodes*, ilApoLima1.1: GC coverage. BlobToolKit GC-coverage plot. Chromosomes are coloured by phylum. Circles are sized in proportion to chromosome length. Histograms show the distribution of chromosome length sum along each axis. An interactive version of this figure is available at https://blobtoolkit.genomehubs.org/view/ilApoLima1.1/dataset/CAMJKE01/blob.

**Figure 4.**
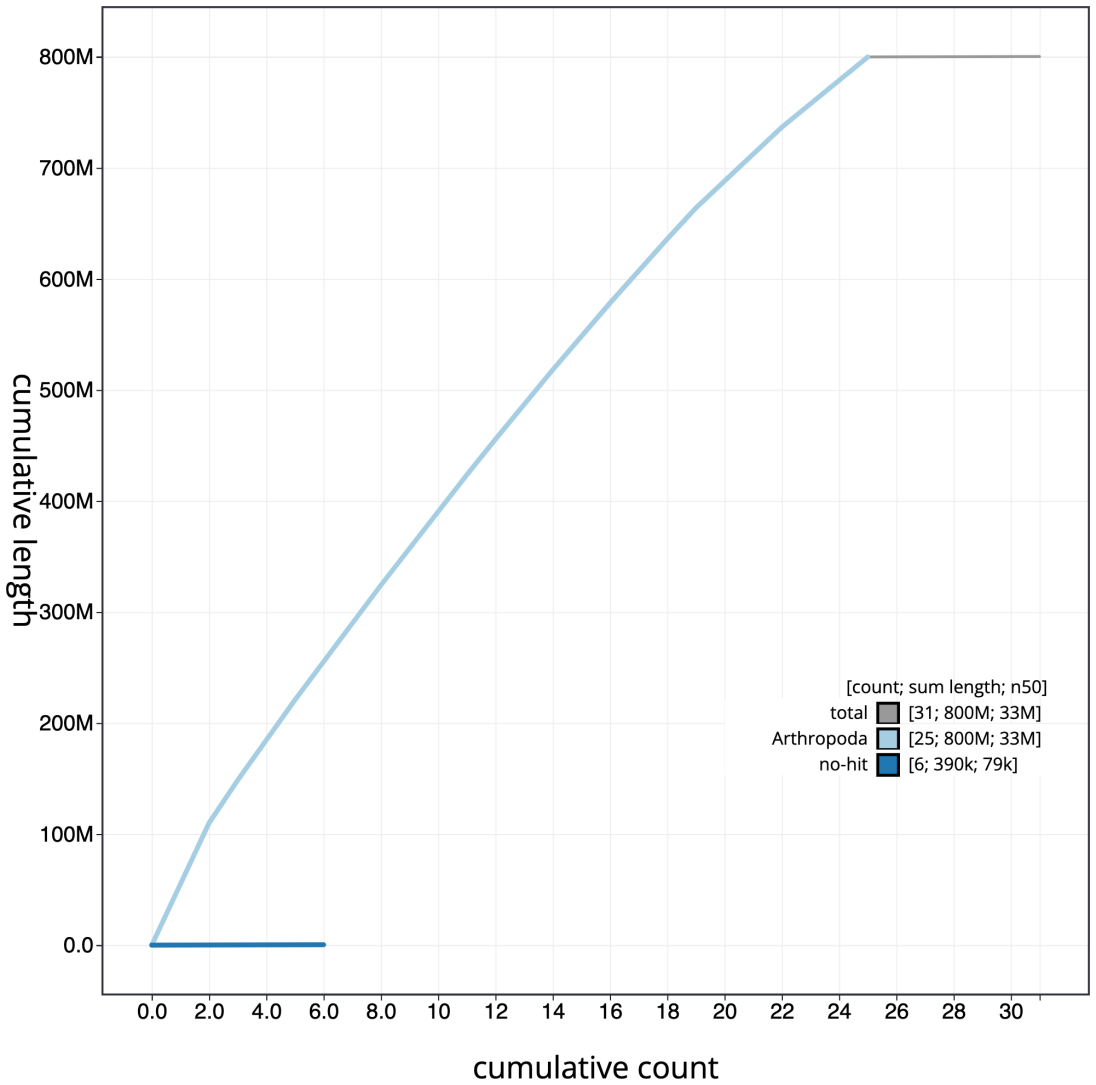
Genome assembly of *Apoda limacodes*, ilApoLima1.1: cumulative sequence. BlobToolKit cumulative sequence plot. The grey line shows cumulative length for all chromosomes. Coloured lines show cumulative lengths of chromosomes assigned to each phylum using the buscogenes taxrule. An interactive version of this figure is available at https://blobtoolkit.genomehubs.org/view/ilApoLima1.1/dataset/CAMJKE01/cumulative.

**Figure 5.**
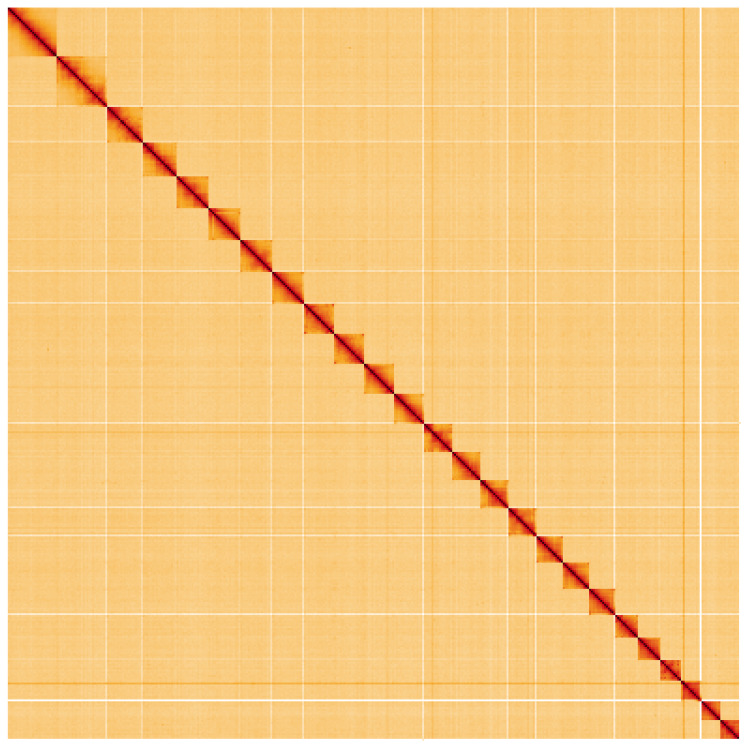
Genome assembly of *Apoda limacodes*, ilApoLima1.1: Hi-C contact map. Hi-C contact map of the ilApoLima1.1 assembly, visualised using HiGlass. Chromosomes are shown in order of size from left to right and top to bottom. An interactive version of this figure may be viewed at https://genome-note-higlass.tol.sanger.ac.uk/l/?d=QnKidS0aRnKlj6qLfctIeQ.

**Table 2.** Chromosomal pseudomolecules in the genome assembly of *Apoda limacodes*, ilApoLima1.

INSDC accession	Chromosome	Size (Mb)	GC%
OX291541.1	1	54.85	35.4
OX291542.1	2	38.48	35.6
OX291543.1	3	36.79	35.7
OX291544.1	4	35.62	35.4
OX291545.1	5	34.64	35.6
OX291546.1	6	34.32	35.7
OX291547.1	7	34.27	35.5
OX291548.1	8	33.04	35.4
OX291549.1	9	33.18	35.7
OX291550.1	10	33.12	35.8
OX291551.1	11	32.08	35.5
OX291552.1	12	31.42	36
OX291553.1	13	31.11	35.4
OX291554.1	14	30.1	35.4
OX291555.1	15	30.04	35.6
OX291556.1	16	29.02	35.3
OX291557.1	17	28.78	35.6
OX291558.1	18	27.89	35.4
OX291559.1	19	24.43	36
OX291560.1	20	24.33	35.6
OX291561.1	21	23.97	35.4
OX291562.1	22	21.25	36.8
OX291563.1	23	21.13	35.5
OX291564.1	24	20.88	35.9
OX291540.1	Z	55.21	34.9
OX291565.1	MT	0.02	18.7
-	-	0.37	40.4

## Methods

### Sample acquisition and nucleic acid extraction

A male *A. limacodes* (ilApoLima1) was collected and identified by Gavin Broad (Natural History Museum). The sample was caught in a garden in Tonbridge, UK (latitude 51.19, longitude 0.29) by using an actinic light and preserved by freezing on dry ice.

DNA was extracted at the Tree of Life laboratory, Wellcome Sanger Institute (WSI). The ilApoLima1 sample was weighed and dissected on dry ice with tissue set aside for Hi-C sequencing. Head and thorax tissue was disrupted using a Nippi Powermasher fitted with a BioMasher pestle. High molecular weight (HMW) DNA was extracted using the Qiagen MagAttract HMW DNA extraction kit. HMW DNA was sheared into an average fragment size of 12–20 kb in a Megaruptor 3 system with speed setting 30. Sheared DNA was purified by solid-phase reversible immobilisation using AMPure PB beads with a 1.8X ratio of beads to sample to remove the shorter fragments and concentrate the DNA sample. The concentration of the sheared and purified DNA was assessed using a Nanodrop spectrophotometer and Qubit Fluorometer and Qubit dsDNA High Sensitivity Assay kit. Fragment size distribution was evaluated by running the sample on the FemtoPulse system.

### Sequencing

Pacific Biosciences HiFi circular consensus DNA sequencing libraries were constructed according to the manufacturers’ instructions. DNA sequencing was performed by the Scientific Operations core at the WSI on Pacific Biosciences SEQUEL II (HiFi). Hi-C data were also generated from tissue of ilApoLima1 using the Arima v2 kit and sequenced on the Illumina NovaSeq 6000 instrument.

### Genome assembly

Assembly was carried out with Hifiasm ( Cheng *et al.*, 2021) and haplotypic duplication was identified and removed with purge_dups ( Guan *et al.*, 2020). The assembly was then scaffolded with Hi-C data ( Rao *et al.*, 2014) using YaHS ( Zhou *et al.*, 2022). The assembly was checked for contamination and corrected using the gEVAL system ( Chow *et al.*, 2016) as described previously ( Howe *et al.*, 2021). Manual curation was performed using gEVAL, HiGlass ( Kerpedjiev *et al.*, 2018) and Pretext ( Harry, 2022). The mitochondrial genome was assembled using MitoHiFi ( Uliano-Silva *et al.*, 2021), which performed annotation using MitoFinder ( Allio *et al.*, 2020). The genome was analysed and BUSCO scores generated within the BlobToolKit environment ( Challis *et al.*, 2020). Table 3 contains a list of all software tool versions used, where appropriate.

**Table 3.** Software tools and versions used.

Software tool	Version	Source
BlobToolKit	3.4.0	Challis *et al.*, 2020
gEVAL	N/A	Chow *et al.*, 2016
Hifiasm	0.16.1-r375	Cheng *et al.*, 2021
HiGlass	1.11.6	Kerpedjiev *et al.*, 2018
MitoHiFi	2	Uliano-Silva *et al.*, 2021
PretextView	0.2	Harry, 2022
purge_dups	1.2.3	Guan *et al.*, 2020
YaHS	yahs-1.1.91eebc2	Zhou *et al.*, 2022

### Ethics/compliance issues

The materials that have contributed to this genome note have been supplied by a Darwin Tree of Life Partner. The submission of materials by a Darwin Tree of Life Partner is subject to the Darwin Tree of Life Project Sampling Code of Practice. By agreeing with and signing up to the Sampling Code of Practice, the Darwin Tree of Life Partner agrees they will meet the legal and ethical requirements and standards set out within this document in respect of all samples acquired for, and supplied to, the Darwin Tree of Life Project. Each transfer of samples is further undertaken according to a Research Collaboration Agreement or Material Transfer Agreement entered into by the Darwin Tree of Life Partner, Genome Research Limited (operating as the Wellcome Sanger Institute), and in some circumstances other Darwin Tree of Life collaborators.

## Data Availability

European Nucleotide Archive: *Apoda limacodes*. Accession number PRJEB54603; https://identifiers.org/ena.embl/PRJEB54603 ( Wellcome Sanger Institute, 2022). The genome sequence is released openly for reuse. The *Apoda limacodes* genome sequencing initiative is part of the Darwin Tree of Life (DToL) project. All raw sequence data and the assembly have been deposited in INSDC databases. The genome will be annotated using available RNA-Seq data and presented through the Ensembl pipeline at the European Bioinformatics Institute. Raw data and assembly accession identifiers are reported in Table 1.

## Author information

Members of the Natural History Museum Genome Acquisition Lab are listed here: https://doi.org/10.5281/zenodo.4790042.

Members of the Darwin Tree of Life Barcoding collective are listed here: https://doi.org/10.5281/zenodo.4893703.

Members of the Wellcome Sanger Institute Tree of Life programme are listed here: https://doi.org/10.5281/zenodo.4783585.

Members of Wellcome Sanger Institute Scientific Operations: DNA Pipelines collective are listed here: https://doi.org/10.5281/zenodo.4790455.

Members of the Tree of Life Core Informatics collective are listed here: https://doi.org/10.5281/zenodo.5013541.

Members of the Darwin Tree of Life Consortium are listed here: https://doi.org/10.5281/zenodo.4783558.
