# Supplementary material for: The genome sequence of the Festoon, Apoda limacodes (Hufnagel, 1766)
Source: Wellcome Open Res. 2023 Jan 12;8:19. [Version 1] doi: 10.12688/wellcomeopenres.18747.1 (PMC10213820; doi:10.12688/wellcomeopenres.18747.1)
Supplement: Peer review history

# Peer review: Reviewer response for version 1

**DOI:** 10.21956/wellcomeopenres.20787.r57505

**Published:** 2023-05-26

**Authors:** Zhaofu Yang

**Affiliations:** 1 Northwest A&F University, Yangling, Shaanxi, China

This paper describes the genome assembly of *Apoda limacode* and provides important information about its size (~800 Mb) and scaffolding (25 chromosomes). The methods for the sequencing and assembly used are appropriate and technically sound. This assembly is the first genome for a species of the family Limacodidae and provides valuable genomic resource for further investigations in the evolutionary pattern of Lepidoptera.

 I suggest add a few reference papers in the second and third sentences in the first paragraph in Background section in case that some readers are interested the taxonomic studies on *Apoda limacode* and Limacodidae.

Are sufficient details of methods and materials provided to allow replication by others?

Yes

Is the rationale for creating the dataset(s) clearly described?

Yes

Are the datasets clearly presented in a useable and accessible format?

Yes

Are the protocols appropriate and is the work technically sound?

Yes

Reviewer Expertise:

Taxonomy, Lepidoptera, Systematics, Molecular phylogeny

I confirm that I have read this submission and believe that I have an appropriate level of expertise to confirm that it is of an acceptable scientific standard.

# Peer review: Reviewer response for version 1

**DOI:** 10.21956/wellcomeopenres.20787.r54121

**Published:** 2023-02-09

**Authors:** Jadranka Rota

**Affiliations:** 1 Biological Museum, Department of Biology, Lund University, Lund, Sweden

Broad et al. present the first genome sequenced from the moth family Limacodidae. Limacodids, also known as slug moths, are a mainly tropical group with close to 2000 described species. They belong to the superfamily Zygaenoidea, which is classified in the large clade Apoditrysia, and this is the least resolved part of the butterfly and moth (Lepidoptera) tree of life. Making the genome of *Apoda limacodes* available is a great step forward in providing more genomic resources for improving our understanding of the evolutionary history of these groups. The genome is of high quality and the text is well written.

 I found one minor typo in the 3 ^rd^ paragraph in the Background section - please remove the parentheses preceding "Shaw & ...":

 “The larvae of *A. limacodes* support a suite of highly specialised parasitoid wasps (e.g., (Shaw & Voogd, 2016) and one…”

 Species authors are missing for *Apoda limacodes* and *Heterogenea asella* in the first paragraph of the Background, but the species author is given further below in the 3 ^rd^ paragraph for the parasitoid *Sphinctus serotinus*. It’d be good to make this consistent in whichever way is preferred by the journal.

 It would be good if the legend to Figure 1 specified that this is a male since there is sexual dimorphism in the species. This is clearly stated in the text, but it would be nice to add it here too.

Are sufficient details of methods and materials provided to allow replication by others?

Yes

Is the rationale for creating the dataset(s) clearly described?

Yes

Are the datasets clearly presented in a useable and accessible format?

Yes

Are the protocols appropriate and is the work technically sound?

Yes

Reviewer Expertise:

Taxonomy, phylogeny, systematics, and evolutionary history of Lepidoptera

I confirm that I have read this submission and believe that I have an appropriate level of expertise to confirm that it is of an acceptable scientific standard.
